# Editorial: Harnessing genomics to revolutionize plant disease management and preservation of soil biodiversity

**DOI:** 10.3389/fmicb.2026.1901274

**Published:** 2026-08-28

**Authors:** Setu Bazie Tagele, Assefa Sintayehu Kassa, Shimeles Tilahun, Workie Anley Zegeye

**Affiliations:** 1 College of Agriculture, Food and Natural Resources, Prairie View A&M University, Prairie View, TX, United States; 2 Department of Plant Sciences, College of Agriculture and Environmental Sciences, University of Gondar, Gondar, Ethiopia; 3 Kangwon, National University, Chuncheon, Republic of Korea; 4 John Innes Centre, Norwich Research Park, Norwich, United Kingdom

**Keywords:** genomics, metagenomics, pathogen effectors, plant disease management, soil biodiversity, sustainable agriculture, transcriptomics

The global population continues to rise and is projected to grow further. Making agricultural productivity more resilient and efficient to keep up with increasing food demand is increasingly challenged by the combined pressures of plant diseases, soil degradation and climate volatility. To address these complex issues, researchers need a deeper understanding of plant-pathogen-microbiome interactions across diverse crop production systems and environmental conditions. Recent advances in high-throughput sequencing (HTS) have transformed our understanding of complex crop production problems, enabling new avenues for exploring biological systems at an unprecedented scale and depth and help translating scientific discoveries into practical agricultural solutions.

Articles in this Research Topic highlight the transformative role of high-throughput sequencing in advancing our understanding of pathogen biology, plant-pathogen interaction, and microbial community dynamics. Together, they demonstrate how sequence-based approaches can reveal microbial community assembly processes, uncover plant pathogen virulence mechanisms, and support the development of sustainable strategies for plant disease management.

A common theme across this Research Topic is the critical role of soil microbiomes in maintaining crop productivity and ecosystem stability. Han et al. investigated how continuous maize cropping (CC) duration influences soil bacterial and fungal community assembly process across a 25-year cultivation period. Their study demonstrated that long-term CC substantially reshapes both bacterial and fungal community structures. With increasing CC duration, bacterial communities became increasingly enriched with dominant taxa, whereas dominant fungal genera exhibited declining trends, suggesting that bacteria and fungi may follow different survival strategies under similar environmental stress. Importantly, their findings demonstrated that prolonged CC drives a shift in microbial community assembly from stochastic to deterministic process, with heterogeneous environmental selection becoming increasingly dominant force. Furthermore, changes in microbial co-occurrence networks suggested that long-term CC reshapes ecological interactions, leading to a shift from competitive to more cooperative associations. These findings provide important ecological insights into how intensive agricultural practices regulate microbial succession and highlight the importance of incorporating microbial community dynamics into sustainable crop production strategies.

The impact of agricultural practices in shaping soil microbial communities has also been illustrated by the work of Tang et al. who investigated the effects of different rice straw-return methods in karst paddy fields. The authors demonstrated that straw incorporation improved soil fertility by altering the soil bacterial community composition, assembly processes, and microbial network structure. Different straw-return methods generated distinct ecological outcomes, highlighting the importance of selecting management practices according to production goals. Rotary tillage straw incorporation with mixing produced the highest rice grain yield by enriching bacterial taxa with nitrogen-fixing capabilities. Their results further revealed that microbial community composition and total nitrogen were the critical factors determining rice yield. This study emphasizes that crop performance is shaped by the combined effects of soil physicochemical properties and microbial ecological processes, reinforcing the importance of microbiome-informed management approaches for sustainable development of karst paddy ecosystems.

Beyond soil microbial ecology, this Research Topic also showcases the transformative role of genomics in understanding plant pathogens and improving disease control. Duan et al. provide the first whole-genome assembly of *Phomopsis asparagi* (*Diaporthe asparagi*), the fungal pathogen responsible for asparagus stem blight. This work marks a significant step forward in understanding the biology of an economically important disease that affects asparagus production worldwide. Through genome annotation and comparative transcriptomics analyses, the authors identified a range of virulence-associated pathways linked to oxidative stress response, reactive oxygen species (ROS) metabolism, cell-wall degradation, and cell dysfunction and programmed cell death. Their investigation of pathogen responses under elevated temperature stress revealed a sophisticated molecular adaptation system involving multiple stress response pathways, proteolysis, ROS generation, DNA repair, and cell wall degradation associated with host colonization. These genomic resources and mechanistic insights establish a valuable theoretical foundation for future research aimed at developing more effective and science-based disease-management strategies.

Advances in understanding pathogen biology are closely linked to innovations in plant resistance breeding. In this regard, Senthilraja et al. review emerging strategies targeting pathogen effector proteins for durable disease resistance. Effectors play a central role in plant-pathogen interactions by manipulating host cellular processes and suppressing immune responses. The authors discuss a range of promising approaches, including susceptibility-gene modification, CRISPR/Cas-based genome editing, RNA interference technologies, effectoromics approaches, and the use of synthetic decoys to enhance immune recognition. By focusing on effector biology, these approaches involve identifying genes to accelerate developing crop varieties with broader and more durable resistance that contribute to cost-effective, eco-friendly and sustainable crop disease management. The review highlights how advances in molecular genetics and biotechnology are reshaping plant protection strategies and contributing to long-term global food security.

Collectively, the studies presented in this Research Topic illustrate the increasingly interconnected nature of genomics, transcriptomics, and metagenomics. The metagenomics studies show how agricultural practices reshape microbial network structure thereby influencing soil ecosystem functioning and crop performance, while the genomics and transcriptomics contributions reveal the molecular mechanisms underlying pathogen virulence factor regulation and pathogenesis-related pathway enrichment.

Several key messages emerge from this body of work. First, sustainable crop disease management cannot be achieved by focusing solely on plant pathogens; it must also account for the broader soil microbiota and their ecological functions. Second, advances in sequencing technologies continue to provide unprecedented insights into both microbiome dynamics and pathogen biology, enabling more precise and informed management strategies. Third, integrating ecological principles with omics-driven innovation offers significant opportunities to enhance crop resilience, maintain soil microbial diversity, and improve agricultural sustainability. Finally, translating these scientific advances into practical applications will require continued collaboration among microbiologists, plant pathologists, breeders, agronomists, and biotechnology researchers.

As agriculture faces the challenges of climate change, resource limitations, and increasing global food demand, the integration of genomics, transcriptomics, and metagenomics will become increasingly important. The contributions assembled in this Research Topic provide valuable insights into the factors shaping soil microbial community assembly and the mechanisms governing pathogen adaptation. Together, these studies demonstrate how omics-driven research can both advance plant disease management through pathogen-targeted strategies and deepen our understanding of how agricultural practices affect soil microbial diversity, thereby contributing to the development of more productive, resilient, and sustainable agricultural systems.

